# Reduced Vastus Medialis/Lateralis EMG Ratio in Volleyballers with Chronic Knee Pain on Sports-Specific Surfaces: A Pilot Study

**DOI:** 10.3390/ijerph19169920

**Published:** 2022-08-11

**Authors:** Christina Frese, Dieter Bubeck, Wilfried Alt

**Affiliations:** Department for Biomechanics and Sportbiology, Institute of Sport and Movement Science, University Stuttgart, Allmandring 28 A, Vaihingen, 70569 Stuttgart, Germany

**Keywords:** stop-landing task, jumping, quadriceps activation, horizontal landing phase, electromyography, sand

## Abstract

Background: Even though chronic knee pain is common in volleyball, neuromuscular imbalance as a potential risk factor has not been investigated in volleyball-specific tasks. The aim of the study was to compare neuromuscular control between healthy and injured players in a clinical jump test and a volleyball-specific jump task in real field conditions. Methods: Six athletes with knee pain and nine controls were included. Surface electromyographic data were recorded from the mm. vastus medialis (VM) and lateralis (VL) of both legs. VM/VL activation ratio was calculated from countermovement jump (CMJ) and volleyball spike indoors and on two beach surfaces. Results: All subjects had pain in the leading leg. Mann–Whitney U Test (M-W-U Test) revealed a significantly lower VM/VL ratio of the leading leg (always affected) of the injured compared with that of the healthy control group for the CMJ and spike jump on all three grounds. Bland–Altman analysis revealed low bias and low difference in standard deviation for the injured leg but high values for the uninvolved leg and healthy controls between tasks and grounds. These results could indicate that neuromuscular control might not adapt too well to different movement tasks and grounds in the injured leg. Conclusion: Athletes with chronic knee pain might have lower VM/VL ratios than controls independent from movement task and ground. Neuromuscular control in injured athletes might be less adaptable to new circumstances. The results of neuromuscular control in laboratory settings might be applicable to field conditions in injured legs but not healthy ones.

## 1. Introduction

The most common overuse knee injuries in volleyball involve patella tendonitis (PT) and patellofemoral pain (PFP) [1]. These pathologies are very serious since current rehabilitation strategies have poor outcomes. Recent research in PFP shows that risk factors are not only multifactorial but that some combinations tend to occur together, forming different subgroups [2,3]. Rehabilitating subjects in accordance with the main risk factors [4] and subgroups considerably improves the outcomes [5]. These results show that knowledge about the main risk factors in one sport or group of patients plays a key role in developing successful rehabilitation and prevention programs. Up until now, there is no consensus on risk factors in volleyball, even though many studies looked at kinetic and kinematic risk factors involving 37 variables since there is a high heterogeneity between study results [6]. The current literature indicates that neuromuscular risk factors could also be relevant for chronic knee pain [7]. It is unclear if neuromuscular risk factors could be the main contributor to volleyball players developing chronic knee pain. To the best of our knowledge, there is no information on this subject matter, especially regarding the data under field conditions.

The most common chronic knee pain conditions involve PT pain and PFP, which, on occasion, may be simultaneously present [8] and occur in all kinds of sports activities [9,10]. Both pathologies seem to occur from similar kinetic and kinematic risk factors [11,12,13,14,15,16,17,18,19,20], also in volleyball-specific movements. Regarding neuromuscular risk factors, a reduced M. vastus medialis (VM)/M. vastus lateralis ratio (VL) [21,22] and delayed onset of VM [23,24] are frequently discussed as risk factors for PFP. Since a lower VM/VL ratio leads to the lateral glide of the patella and patellar rotation [22], which creates stress on the tendon in simulation experiments [19], it can be assumed that the patellar movement of the inferior pole leads to the further inconvenient distribution of the patellar tendon strain and provoke PT pain. Therefore, it seems reasonable to consider neuromuscular risk factors in both pathologies together. However, neuromuscular risk factors might depend on gender since females with PFP have less than 10 degrees of hip abduction and more than 10 degrees of tibial internal rotation, and males have a reduced knee flexion (<20 degrees) in gait [16]. Similar results were obtained by Willy et al. for running [25]. Additionally, female volleyball athletes show different movement characteristics than males [26,27]. Nevertheless, all studies concerning PT pain in volleyball athletes included solely males, as shown in two reviews [6,28]. Additionally, there are no studies on risk factors in young volleyball athletes, since all investigations included adults predominantly in the age of 20–30 years [11,28,29,30,31,32]. Therefore, studies examining neuromuscular control in young female athletes are necessary for further insights. 

In regard to neuromuscular risk factors, movement task [33] and surface [34] might be crucial factors since volleyball can be performed indoors on hard surfaces and outdoors on sand surfaces. Both disciplines are part of the Olympic games [35,36]. Information on neuromuscular control in spike jumps in healthy and injured athletes is not available, neither on hard surfaces such as in a hall nor in sand conditions. Therefore, there is no information if the data from laboratory settings, mostly from a CMJ or squat jump [37], are applicable to spike jumps under field conditions. The current literature indicates that those data might not be applicable, since the VM/VL ratio might decrease with increasing demand on movement tasks in normalized [33,38,39] and non-normalized data [38]. Further studies prove that neuromuscular and biomechanical adaptations occur on different surfaces [40,41,42]. Those surfaces influence the rate of overuse injuries [42,43,44]. Hence, the sports-specific surfaces should be taken into consideration [45]. Another aspect of professional beach volleyball is that athletes all around the world play on different sand surfaces varying in their structure, especially concerning grain size [46]. Only Peikenkamp [47] investigated the effect of sand properties on neuromuscular mechanics, which also involved the mm. vastus medialis (VM) and lateralis (VL) activity on the dry and wet sand. Peikenkamp et al. [47] concluded that sand type influences neuromuscular control. Consequently, different sand structures and indoor surfaces may influence neuromuscular control. This could be especially relevant for athletes with PT pain since they might be less capable to adapt neuromuscular control in a jump task, as was already proven in kinematic aspects [48] in order to decrease tendon loads in comparison to healthy subjects. 

In volleyball, the spike jump is the most forceful, dynamic, and complex movement, involving the highest generation of power [49]. The uniqueness of the approach phase in the spike jump is that the leading leg undergoes a “horizontal landing” phase, in which it transfers the horizontal velocity into jump height [49]. In this example, the amount of stress on the knee is even higher than in the landing after the jump. Consequently, the horizontal landing phase is considered to play a major role in the development of knee pain [49,50]. Therefore, we assume that the spike jump is the most suitable movement for determining risk factors for chronic knee pain in female athletes. The CMJ is one of the most widely [37] used jumps in performance testing and, therefore, a good counterpart to spike jumps.

Knowledge about neuromuscular gender-specific risk factors in athletes with and without chronic knee pain in different field conditions can contribute to specified diagnostic tests and prevention programs. For coaches, it is essential to understand the influence of the surface on neuromuscular control, in order to create training programs with appropriate load increase when changing from indoor to beach and vice versa or between sand surfaces. 

Consequently, the first aim of the study was to investigate neuromuscular imbalances in young female volleyball athletes with and without chronic knee pain in two different settings: in a basic (CMJ) and a sports-specific jump task (spike jump) and on different sports-specific surfaces. These different surfaces include one hard and two sand surfaces. Secondly, we aimed to gain a better understanding of the validity of clinical tests (CMJ) in the laboratory for applicability in field situations (spike).

## 2. Materials and Methods

A pilot study on volleyball athletes of the junior squad at the Olympic Training Center of Stuttgart was carried out. The study was cross-sectional comparing athletes with and without anterior knee pain. The study design, data collection, and data analysis were carried out by the same two investigators (a research assistant (MSc Biomechanics in Movement analysis) and a research advisor (PhD biomechanics)). Inclusion criteria were unilateral localized or diffuse anterior knee pain [51] in the last four weeks in volleyball. Most studies used the presence of pain in at least two of the following functional activities as inclusion criteria: prolonged sitting, climbing up and down stairs, squatting, kneeling, running, and jumping. Since volleyball training includes running and jumping, and all subjects perform the same sport, the inclusion criterion was knee pain while performing volleyball. One further inclusion criterion was regular participation in volleyball training. Exclusion criteria were acute injuries of the back or the lower limb during the last six months. All subjects were briefed on the details of the study and gave their written consent prior to participation. In the case of minors, parents signed the confirmation prior to testing. The study protocol was approved by the local ethic committee of the University of Stuttgart (Az. 21-044).

### 2.1. Study Protocol

Firstly, EMG electrodes and foot insoles were placed on the subject bilaterally. The VM and VL EMG activity was bilaterally measured, according to recommendations of the SENIAM [52]. Data were recorded at 1500 Hz with Noraxon 2400 Telemyo G2 (Noraxon Corporate, Scottsdale, AZ, USA). Plantar pressure insoles were attached to the foot (T&T Medizintechnik GmBH, Schönefeld, Germany) to detect the horizontal landing phase. The validity and reliability of this equipment in comparison to a force plate were proven by Koch et al. [53]. Indoors, the insoles were put into the shoe, whereas in both sand conditions, the insoles were fixed with an adhesive sock.

Following preparation, the subjects warmed up for the actual measurements. Measurements were performed at three different locations: indoors (referred to as a hard surface in the following), sand surface 1 (rated to be softer in structure than sand surface 2 by coaches and staff), and sand surface 2. The surfaces were randomly used, while the jump order remained stable. The 15 min warm-up of legs and shoulders consisted of running, throwing, and striking drills.

Subjects were asked to stand in an upright position, with their hands on their hips while jumping as high as possible without using an arm swing for the CMJ. The test–retest reliability between days of non-normalized integrated EMG of RF (ICC = 0.88) and VM (ICC = 0.7) for CMJ was reported as high [54]. Additionally, the test–retest reliability of non-normalized VM and VL and the VM/VL IEMG ratio in isometric contractions is considered excellent (ICCs > 0.86) [55]. The significance level was set to α < 0.05. Then, athletes needed to carry out a complex spiking task that simulated training situations. Even though there are no studies investigating EMG reliability in the takeoff phase in a stop-landing task, there is one study reporting high reliability in VL (ICC = 0.92) and VM (ICC = 0.86) activity in landing [56]. The horizontal landing phase consists of two parts. In the first part, the leading leg breaks the force, and in the second phase, both legs execute the propulsion upwards. Since the IEMG is reliable in landing after a jump, we assume that it is also reliable in the breaking phase of the horizontal landing phase. Similarly, since the IEMG is also reliable in the takeoff phase of a CMJ, we assume that the IEMG in the propulsion phase of the takeoff, which is similar to the propulsion phase in CMJ, is also reliable. When a staff member threw the ball into the air, another staff member on the opposite field ran from the middle to the right or left side at random. Athletes needed to spike to the counter-direction. Five to ten testing trials were performed, until the thrower and the athlete both agreed on having good timing. Three to five extra trials were recorded, depending on the repetitions needed to gain three successful trials. As soon as the first measurement series was acquired, everything was repeated on the other surfaces.

### 2.2. Data Processing

Jumps were separated with the Noraxon^®^ software MR 3.12 (Scottsdale, Arizona) by detecting vertical ground reaction force. The horizontal landing phase detection, as well as the VM and VL activation, were calculated with a customized MATLAB script (MathWorks, Inc., Natick, MA, USA). The beginning of the jump phase in the CMJ was defined as the point in time at which the vertical ground reaction force (vGRF) decreased three standard deviations below the body weight. It ended when the summarized vGRF of both legs was less than 10 newtons. In the spike horizontal, the landing phase was defined between touchdown (vGRF > 10 n) and takeoff (vGRF < 10 n) for the leading leg. Raw EMG signals were rectified and smoothed using a 50 ms moving average window. The integrated EMG activity was calculated for the VM and VL between the starting point and the end point, detected by the insoles. In the calculated ratio, the VM was expressed in relation to the VL activity (VM/VL ratio = IEMG VM/IEMG VL), in accordance with Souza et al. [38]. We chose not to normalize the data to the MVC, since Souza et al. concluded from their investigation that normalizing activation to the MVC may have resulted in the inability to detect differences among subgroups. The neuromuscular imbalance already present in maximal voluntary contraction in some subjects [33,38] could bias results. Other authors have also used non-normalized IEMG data in cross-sectional study designs with jump tasks for different research questions [57]. Therefore, the non-normalized IEMG should be taken into consideration for detecting intra-individual imbalances in the VM/VL ratio.

### 2.3. Statistical Analysis

The first jump was used for the statistical analysis of the EMG data. Data are presented as box plots. Due to the low sample size, the Mann–Whitney U test was used to determine group differences. The binary-coded independent variable was the injury status of the leading leg (yes or no). Differences between jump tasks and surfaces for the leading leg were determined with the Wilcoxon signed-rank test. Due to the low sample size (4–6 athletes), an intra-individual comparison was made. Statistical tests were not applied. The dependent variable was always the non-normalized VM/VL ratio. The similarities of the VM/VL ratios between jump conditions and surfaces were tested with Spearman’s correlations. The interpretation of correlation coefficients was based on the works by Zou et al., who proposed the following order: 0.2 weakly positive; 0.5 moderately positive; 0.7 strongly positive, and 1.0 perfectly positive [58]. The differences between both jumps, between the leading and providing leg in the injured group, and between surfaces for the spike jump were tested with a Bland–Altmann analysis.

## 3. Results

Eight athletes were excluded from further analysis due to several reasons:

- Four athletes could not attend for measurement due to medical circumstances;
- From two athletes, data could be taken but were excluded from analysis due to measurement problems;
- From two further athletes, data could be taken but were excluded from the main analysis since those two athletes had bilateral knee pain. All the data are separately listed in the Supplementary Materials.

Therefore, 15 female athletes (healthy: 9, injured: 6) with unilateral knee pain were included. The 13 youngest athletes (14–16) played indoors most of the year (September–April) and beach volleyball in summer (May–August). At a later stage, approximately at 18 years of age, they specialize in indoor or beach volleyball. The two remaining U-21 athletes have been specializing in beach volleyball for 2–3 years (they do not play indoors anymore) but also had high levels of experience in indoor volleyball from former years (about 5 years). Most of the athletes started playing between the ages of 10 and 12. Hours actively played ranged from 6 to 15 h per week. All athletes were involved at national and international levels.

Group characteristics are displayed in Table 1. The average age, weight, and height of both groups were not significantly different. Regarding the playing position, beach volleyball athletes, setters, and liberos do not have knee pain. In this pilot study, middle hitters and foremost outside hitters had unilateral chronic knee pain on their leading limb. Since all players were still able to perform their sport in training (2–3 h) and competitive matches for several hours (1–4 h) on a training day, the pain level was estimated between 2 and 6 on a VAS scale in volleyball. The number of jumps performed in this setup is far lower than the number of jumps in training and match.

### 3.1. Kinetics of the Leading and Providing Leg in Spike Jumps

In the horizontal landing phase, the leading leg had a significantly higher impulse (Ø 3.5 Ns/kg ± 0.95 Ns/kg) than the providing leg (Ø 1.6 Ns/kg ± 0.59 Ns/kg) in injured and healthy athletes. In the landing phase, after the jump, the providing leg had a higher impulse (Ø 5.0 ± 2.69 Ns/kg) than the leading leg (Ø 1.42 ± 1.49 Ns/kg), but this was not always significant. No group differences were detected. Charts with more data containing means, standard deviations, and individual datasets are provided in Appendix S1.

### 3.2. Group Differences in VM/VL Ratios

**Figure 1:**
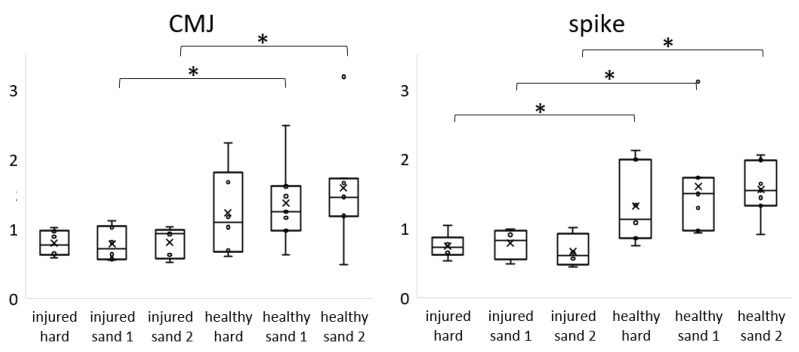
Box plots of VM/VL ratios of athletes with and without chronic knee pain on the leading leg. Ratios are displayed for all three surfaces: hard surface, sand 1, and sand 2. Significant group differences (α < 0.05) are marked with *. Individual VM/VL ratios are displayed as dots.

The box plots of VM/VL ratios of the leading leg are presented in Figure 1. All the raw data are available in Appendix S2 in the Supplementary Materials. The median (line in Figure 1) and mean (crosses in Figure 1) values of injured athletes were roughly 0.8 ± 0.2 and for healthy athletes roughly 1.3 ± 0.5. The individual results are presented in Appendix S2 in which those of uni- and bilaterally injured athletes are listed. There was low variability in the means, medians, and standard deviations observed between surfaces for athletes with chronic knee pain, whereas higher variability in means and standard deviations were noted for healthy athletes in both jumps. Significant differences were found between the leading legs of injured and healthy athletes for all spike jumps, and CMJs in sand 1 and sand 2 for α-level < 0.05. No significant group difference was found between CMJs on the hard surface. 

The median values and interquartile ranges are also displayed in Table 2.

### 3.3. Spearman’s Correlation of Jump Tasks and Surfaces

There was a significant relationship (*p* < 0.01) for neuromuscular control (VM/VL ratio) between jump tasks and between grounds (Table 3).

Raw values and figures displaying the VM/VL ratios between jump tasks and surfaces are available in Appendix S3 in the Supplementary Materials. The correlation between CMJ and spike jump was highest on sand 2 and lowest on sand 1. The correlation between surfaces was highest for the hard-sand 2 conditions and lowest for the sand 1-sand 2 conditions. There were no significant differences in the VM/VL ratios between jump tasks and surfaces. Athletes with and without chronic knee pain were included in this analysis. 

### 3.4. Bland–Altman Analysis of Intra-Individual Differences, Jump Tasks, and Surfaces

The intra-individual analysis revealed that VM/VL ratios were almost equal for both jumps on the hard surface. Furthermore, the VM/VL ratios of the uninvolved providing leg were higher in both sand conditions for CMJs (sand 1 = 0.15; sand 2 = 0.25) and for spike jumps (sand 1 = +0.59; sand 2 = 0.6). Raw values and Bland–Altman plot are available in Appendix S4 in the Supplementary Materials. The most important results of Bland–Altman analysis are displayed in Table 4.

The jump task analysis was separately performed for the injured and healthy groups. We considered the CMJ as the gold standard and, therefore, compared the spike jump to the former. There was no bias between CMJs and spike jumps on the hard surface (−0.01) and sand 1 (−0.01) for the injured leg. VM/VL ratios tended to be lower for spike jumps with the injured leg in sand condition 2 (−0.11). Differences in standard deviations ranged between 0.09 and 0.24. In contrast, the VM/VL ratios in healthy athletes were higher in spike jumps on the hard surface (+0.13) and sand 1 (+0.09) than those found in CMJs. However, the ratio was lower in sand 2 (−0.27). The differences in standard deviations in the healthy group were higher (between 0.15 and 0.45) than those in the injured group. There were not any significant differences between jump tasks. 

As for surfaces, we considered the hard surface the gold standard, since clinical diagnostic jump tests are normally performed indoors on hard surfaces. Examining healthy and injured participants independently, the injured group had almost no difference in mean values (hard surface − sand 1 = 0.02; hard surface − sand 2 = −0.08; sand 1 − sand 2 = 0.00), and standard deviations were small to moderate, with 0.15, 0.05 and 0.11, respectively. The VM/VL ratios of the leading leg in the healthy group were higher for sand 1 (+0.2) and sand 2 (+0.15) than for the hard surface and higher in sand 2 (+0.14) than in sand 1. Additionally, there were higher differences between standard deviations of 0.48, 0.17, and 0.4, respectively, than those observed for the leading leg of injured athletes.

## 4. Discussion

The first aim of the study was to compare neuromuscular imbalances in the horizontal landing phase in young female athletes with and without chronic knee pain, performing a CMJ and spike jump specific to volleyball. We assume that this phase is critical in this cohort since all subjects had unilateral pain in their leading leg, and the horizontal landing phase generates high forces predominantly on the leading leg (Ø 3.5 ± 0.95 Ns/kg), compared with the providing leg (Ø 1.6 Ns/kg ± 0.59 Ns/kg). This was also shown in other studies [49]. Landing after a jump predominantly occurs on either both legs or the providing leg. We drew that conclusion based on our kinetic data (Ø leading leg: 1.42 ± 1.49 Ns/kg versus Ø providing leg 5.0 ± 2.69); in addition, it was also shown in previous studies [59]. The exact values are displayed in the Supplementary Materials in Appendix S1. In contrast to Richards et al., we could not find any group differences [11], but our findings are in line with the results of other studies [13,31,32]. With reference to our results, we concluded that VM/VL ratios were lower in athletes with chronic knee pain in the involved limb, independently from the type of jump, surface, and origin of the knee pain in the horizontal landing phase; however, there was no crossover effect on the uninvolved side. The VM/VL EMG ratios of healthy athletes ranged between 1.24 and 1.61 in our study, which is in line with previous results by Jafarnezhadgero, reporting ratios ranging from 1.18 to 1.65 [60]. In contrast, the VM/VL ratios in the leading leg (Ø_CMJ_ = 0.64; Ø_spike_ = 0.65) and the providing leg (Ø_CMJ_ = 0.52; Ø_spike_ = 0.69) in the two excluded athletes with bilateral pain were reduced on all surfaces and jumps and even lower in the injured leading limb of unilaterally injured athletes (Ø_CMJ_ = 0.74; Ø_spike_ = 0.80). We concluded that neuromuscular misbalance in the injured leg, regardless of whether it was the leading or providing leg, was present in all kinds of activities and had no crosstalk effect on the healthy side. All in all, our results support previous findings involving squat exercises [33], single-leg squats [61], ascending and descending stairs [62], and isometric knee extension tasks [38]. Even though many studies focused on the neuromuscular imbalance of the VM/VL in patients with PFP [21,22], there are no current studies we know of that examine neuromuscular control in PT [23]. Therefore, this study is the first to show that neuromuscular imbalances may not only exist in athletes with PFP but also in those with PT pain and that such imbalances might only exist on the involved side. These results are in line with our expectations since both knee pathologies share many kinetic and kinematic risk factors [11,12,13,14,15,16,19,20] and occur both in sports activities that involve stop-landing tasks and running [9,10]. Based on the results by Souza, we decided not to normalize the EMG, since neuromuscular disbalance also exists in maximal voluntary contraction in some subjects [33,38] and may have resulted in the inability to detect differences among the subgroups. Therefore, the non-normalized IEMG should be considered for detecting imbalances in the VM/VL ratio. Furthermore, the reliability of the non-normalized EMG data of quadriceps (ICC = 0.8) [63] and the VM (ICC = 0.7) [54] in CMJs was proven to be high.

Our results may not be completely applicable to bilateral pain, the male athlete, or older subjects. In contrast to unilateral pain, bilateral pain or pain on the providing leg would be more likely to result from landing after a jump, as shown by Richards [11]. For example, knee flexion angle in landing after a jump predicted knee pain in 10 out of 10 cases correctly for the providing knee. For the leading leg, however, the ground reaction force and its time derivative in the takeoff phase are significant predictors (8 of 10 cases). These results indicate that the kinetic risk factors might differ between pain on the leading leg and the providing leg, and bilateral pain. In contrast to our study, four of six athletes with PT had bilateral pain [11]. The high percentage of bilateral pain in the male cohort and predominantly unilateral pain on the leading leg in our female cohort can support the assumption that risk factors might also differ between genders in volleyball [16,26]. For instance, in a prospective study design, Boling et al. demonstrated that the risk factors for females with PFP in gait included less than 10 degrees of hip abduction and more than 10 degrees of tibial internal rotation, and for males, a reduced knee flexion (<20 degrees) [16]. Similar results were obtained by Willy et al. in running [25]. Since all volleyball-specific studies solely included males, as shown in two reviews [6,28], and male and female volleyball athletes are distinct in their movement characteristics [26], our assumption that the takeoff phase is the critical phase might not be applicable to the male athlete. In regard to the risk factors in neuromuscular control, a reduced VM/VL ratio might also be applicable to male and female athletes with bilateral pain since both bilaterally injured athletes (1 m/1 f) had reduced ratios on either side. Furthermore, current studies solely included male adults, predominantly between the ages of 20 and 30 years [11,29,30,31,32], even though the incidence of PT and PFP is also high [64,65] in young athletes. Our results indicate that VM/VL disbalances already exist in younger athletes.

The second aim was to gain a better understanding of the validity of clinical tests in laboratory settings for applicability in field situations. Recent research indicates that in-lab kinematics can differ greatly from in-field kinematics (*p* < 0.044). It is not clear if those differences are also applicable to neuromuscular activity [40]. This includes applicability for jump tasks and surfaces. The non-normalized EMG seemed to be most suitable for these comparisons since the subjects acted as their own controls, and electrode positions were not changed [55,66].

Based on the literature, the VM/VL ratio may decrease with the increasing demand on movement tasks. For instance, the normalized VM/VL ratios were lower in complex functional tasks [33,38] than in isometric, isolated extension tasks. Additionally, subjects with reduced VM/VL ratios in isometric tasks had reduced VM/VL ratios in double-legged squats but not vice versa [33]. Even though there are no current studies on the VM/VL ratio in different jumps, Kopper et al. reported that VL activation in a DJ (*p* = 0.003) and CMJ (*p* = 0.012) was significantly higher than that in a squat jump. This suggests neuromuscular adaptations according to jump type [67]. Consequently, the VM/VL ratio may be dependent on the movement task. We cannot completely confirm these findings since the neuromuscular imbalance in the injured leg was the same in the spike jump as the imbalance found in CMJ on the hard surface (difference in mean = −0.01) and sand 1 (difference in mean = −0.01). Only in sand 2 was the VM/VL ratio in the spike jump lower than that in CMJ (difference in mean = −0.11). On the other hand, looking at the neuromuscular response of the leading limb of healthy athletes (hard = 0.13; sand 1 = 0.09; sand 2= −0.27), we concluded that neuromuscular adaptions occur. Comparing the injured and uninjured legs of the unilaterally injured athletes, it seemed that the uninjured leg also increased the VM/VL ratio with task complexity, since the differences in the mean values between the injured and uninjured leg were higher in spike jumps (hard = 0.03; sand 1 = 0.59; sand 2 = 0.60) than in CMJs (hard = 0.1; sand 1 = 0.15; sand 2 = 0.25). We concluded that the neuromuscular response might be reduced in the injured compared with that in the healthy legs. The same applied when changing between surfaces. In contrast to the VM/VL ratio of the injured limb, which had low differences in the mean values between surfaces (hard surface − sand 1 = 0.02; hard surface − sand 2 = −0.08; sand 1 − sand 2 = 0.00), the healthy athletes had higher ones (hard surface − sand 1 = 0.20; hard surface − sand 2 = 0.15; sand 1 − sand 2 = 0.14). Again, the injured limb presented itself with a lower standard deviation of difference (<0.15) than the healthy one (<0.48). Comparing the injured and uninjured legs of the unilaterally injured athletes again, it seemed that the uninjured leg, like the healthy legs, increased the VM/VL ratio in sand conditions since the mean differences between both legs in sand conditions in CMJs (sand 1 = 0.15; sand 2 = 0.25) were higher than on that on the hard surface (hard = 0.1). The same applied for the spike jumps (sand 1 = 0.59; sand 2 = 0.6 vs. hard = 0.03). These results are in line with the EMG data taken in running on hard surfaces and sand. Jafarnezhadgero investigated neuromuscular control in healthy individuals with overpronated feet and normal feet. The VM/VL ratio increased on sand for the overpronated group (hard surface = 1.21 vs. sand = 1.65) and stayed the same for the clinically unremarkable foot group (hard surface = 1.18 vs. sand = 1.19) [60]. The results of the bilaterally injured athletes support our assumptions because the VM/VL ratio increased neither in the injured leading leg nor in the injured providing leg, with task complexity or in the sand conditions. Since the VM/VL ratio considerably varied between surfaces and jump tasks for the leading leg of healthy athletes and the healthy providing leg of unilaterally injured athletes, we assume that neuromuscular adaptations to surface and task are physiological. Therefore, neuromuscular control in the injured leg might be less capable to adjust to different movement strategies. These assumptions are supported by several other works: Edwards et al. showed that athletes with patellar tendon abnormality are also less capable to adapt their kinematics in a fatigued state, in order to reduce tendon loads, in contrast to controls [48]. Recent research by Tenriwulan et al. indicates that runners with a current chronic overuse injury or with a history of lower limb injury have lower gait variability in gait kinetics than healthy runners [68]. Hence, injuries may result in reduced variability of kinematics [48], kinetics [68], and neuromuscular activity. More research on the correlation between movement variability and risk for future injuries is necessary to solve this gap. 

Due to the low mean differences and small differences in standard deviations in the involved limb for surfaces, we conclude that neuromuscular diagnostics on hard surfaces, which is typical in laboratory settings with sports-specific jumps, might be a good tool to diagnose neuromuscular disbalances in injured athletes on sports-specific surfaces such as sand. Since there were also a low mean difference and standard deviation between jump tasks, CMJs instead of spike jumps may be suitable to detect neuromuscular disbalances. Due to higher neuromuscular variation in healthy legs, this may not be applicable to healthy controls.

The study is limited because only six unilaterally injured and nine healthy athletes participated. Results and discussion were extended by two bilaterally injured athletes one of whom was a male athlete. The study group was not completely homogenous, since two athletes solely played beach volleyball for the last two to three years, and the others played indoor and beach volleyball. Nevertheless, since the VM/VL ratios of the two beach players and the male athlete lay within the norm of the two different groups, we do not expect a bias. Due to the low sample size, it cannot be clarified whether these results are general correlations or individual ones. This study could only show tendencies and compare them with the existing literature. Additionally, most of the players were female, so these results need to be confirmed for male players. There might be a good chance that male athletes also suffer from this disbalance since the only male bilaterally injured athlete had reduced VM/VL ratios on both sides as well. However, tiny numbers are a general problem in studies related to performance sports. The reason for only including athletes of national and international levels was to exclude acute adaptive effects by inexperienced athletes on the different surfaces. Another limitation was that we did not gather information about pain severity on a VAS scale. Pain severity might influence study results. Since all players could still engage regularly in volleyball training, we assume that the VAS score was rather low. Athletes with higher VAS scores might show even higher misbalances. Future studies should include volleyball players with higher VAS scores and subjective reduction in playing performance to gather information on the correlation between subjective pain level and neuromuscular control deficits. Furthermore, we did not clinically distinguish between PT and PFP. This could have an effect on study results. Future studies should include and note the number of athletes with PT and PFP. One further limitation is that it was not possible to calculate the VM/VL force ratio from the EMG activity, which is probably the main contributor to patellar movement and patellar tendon strain. Additionally, it is unclear if the non-normalized EMG data contained fewer errors than the normalized ones for group comparison [38]. However, it is considered valid for comparisons between surfaces and jumps since subjects serve as their own controls [66]. The last limitation was exposure to weather. Extreme heat on some days promoted sweating and, therefore, sometimes the malfunction of electrodes. They were replaced when artifacts could be seen in the EMG. Even though this is not desirable, it is not preventable when measuring outside on the field in extreme conditions. Since outdoor measurements are more prone to complications, most of the studies are carried out in laboratory settings. Nevertheless, our results showed that field testing can reveal important information and should be performed more often since there is so little knowledge concerning field testing. 

## 5. Conclusions

In this pilot study, we examined neuromuscular activity in healthy and injured athletes in basic diagnostic jumps and sports-specific jumps on sports-specific surfaces for the first time. This pilot study revealed the following findings:

1. Neuromuscular alterations such as the VM/VL ratio might be an important risk factor in female volleyball players with knee pain.
2. Female athletes could have a higher tendency for unilateral knee pain on the leading limb. Due to high forces on the leading limb in the horizontal landing phase and low forces in the landing phase after the jump, the horizontal landing phase might be more relevant for female athletes with unilateral knee pain on the leading limb.
3. The neuromuscular control of the injured leg could be less adaptable to jump tasks and surfaces. Therefore, basic jump tasks on hard surfaces might be good predictors for neuromuscular control in sports-specific tasks on sports-specific surfaces. This might not be applicable to healthy athletes.

For clinical application, these results imply that doctors and physiotherapists should look for neuromuscular deficits in injured volleyball players and that basic diagnostic jumps might be appropriate to measure these deficits. An additional focus on the biomechanics in the horizontal landing phase might be appropriate when investigating volleyball athletes with unilateral knee pain on the leading limb. 

In research contexts, the results of this pilot study can provide information for a more comprehensive approach in regard to clarification of chronic knee pain, since “comparison of mechanics across a variety of tasks within the same cohort […] help define tasks likely to reveal abnormal mechanics”, considered essential in the third patellofemoral research retreat [69]. Even though we cannot derive final answers from our data, our results provide a foundation for further biomechanical research.

Based on our data, we suggest that future studies should not only consider kinetic and kinematic risk factors but should also more closely examine the neuromuscular risk factors in female and male athletes in larger populations. Based on our data and results, the literature should consider differences in risk factors for unilateral pain on the leading and providing leg, as well as bilateral pain. Examining those athletes together could blur different risk factors. This might also be applicable to gender. Finally, our results imply that the applicability of data between tasks and surfaces might depend on healthy and injured athletes. Therefore, future studies should examine this issue on healthy and injured athletes separately. 

## Figures and Tables

**Table 1:** Subject characteristics such as age, weight, and height of the healthy and injured groups are expressed in means and standard deviations. Specialization in regard to sports activity type (indoor versus beach) and playing position is expressed in categorical variables for “overall” and in percentages for “healthy” and “injured” in relation to “overall”.

	Overall	Healthy	Injured
Age (years)	15.2 ± 1.9	15.5 ± 2.4	14.7 ± 0.5
Weight (kg)	63.8 ± 5.7	64.0 ± 7.0	63.5 ± 3.8
Height (m)	1.78 ± 0.1	1.78 ± 0.1	1.79 ± 0.04
Gender	female	female	female
Beach	2	2	0
Outside hitter	6	2	4
Middle hitter	5	3	2
Setter	1	1	0
Libero	2	2	0

**Table 2:** Median values and interquartile ranges in CMJs and spike jumps for the leading limb of healthy and unilaterally injured athletes. All unilaterally injured athletes had pain in their leading limb.

		Injured			Healthy		
		Hard	Sand 1	Sand 2	Hard	Sand 1	Sand 2
CMJ	median	0.77	0.72	0.78	1.10	1.25	1.46
	IQR	0.35	0.48	0.41	1.14	0.63	0.54
spike	median	0.73	0.83	0.61	1.13	1.50	1.55
	IQR	0.18	0.24	0.22	0.54	0.74	0.39

**Table 3:** Spearman’s correlation coefficients of VM/VL ratios for jump tasks and surfaces. Correlations of VM/VL ratios between CMJs and spike jumps on the three various surfaces are displayed in the upper row. Correlations of VM/VL ratios in spike jumps between the three surfaces are displayed in the lower row. * indicates significant correlation with *p* < 0.01.

**CMJ vs. Spike**	**Hard**	**Sand 1**	**Sand 2**
	0.88 *	0.85 *	0.91 *
**Surfaces**	**Hard-Sand 1**	**Hard-Sand 2**	**Sand 1-Sand 2**
	0.91 *	0.97 *	0.69

**Table 4:** Differences in means, standard deviations, and upper and lower limits of 95% confidence intervals of Bland–Altman analysis for intra-individual differences, jump tasks, and surfaces. The upper table part resents the difference in VM/VL ratios between the uninjured and injured legs of unilaterally injured athletes. This comparison was made for the CMJ and spike jump. VM/VL ratio of the uninjured leg, especially in the spike jump, was higher than in the injured leg. The middle part presents the difference in VM/VL ratios between the counter-movement jump (CMJ) and spike jump for the leading leg and the lower part between surfaces. In both cases, the data were split into the leading leg of injured and healthy groups. Differences in means, standard deviations, and confidence intervals were much lower for the leading leg in the injured group compared with those of the healthy group for jump tasks and surfaces.

**Intra-individuell (mean difference between uninjured and injured)**			**Hard**	**Sand 1**	**Sand 2**
	**CMJ**	n	6	6	6
		mean difference	0.1	0.15	0.25
		standard deviation	0.403	0.474	0.475
		CI-upper	0.88	1.07	1.17
		CI-lower	−0.69	−0.78	−0.68
	**Spike**	n	6	4	4
		mean difference	0.03	0.59	0.6
		standard deviation	0.344	0.479	0.587
		CI-upper	0.7	1.52	1.75
		CI-lower	−0.64	−0.35	−0.54
**Jump tasks (mean difference between CMJ and spike)**			**Hard**	**Sand 1**	**Sand 2**
	**Injured leg**	n	5	5	4
		mean difference	−0.01	−0.01	−0.11
		standard deviation	0.09	0.24	0.11
		CI-upper	0.15	0.46	0.11
		CI-lower	−0.17	−0.49	−0.32
	**Healthy leg**	n	6	6	5
		mean difference	0.13	0.09	−0.27
		standard deviation	0.15	0.39	0.45
		CI-upper	0.43	0.84	0.61
		CI-lower	−0.16	−0.67	−1.14
**Surface (mean difference between surface 1 and surface 2)**	**Injured leg**		**Hard-Sand 1**	**Hard-Sand 2**	**Sand 1-Sand 2**
		n	5	4	3
		difference-mean	0.02	−0.08	0.00
		standard deviation	0.15	0.05	0.11
		CI-upper	0.32	−0.01	0.16
		CI-lower	−0.28	−0.15	−0.16
	**Healthy leg**	n	7	6	5
		difference-mean	0.20	0.15	0.14
		standard deviation	0.48	0.17	0.40
		CI-upper	1.15	0.48	0.94
		CI-lower	−0.75	−0.19	− 0.65

## Data Availability

The data presented in this study are openly available in FigShare at https://doi.org/10.6084/m9.figshare.19229484 (accessed on 29 July 2022).

## Supplementary Materials

The following supporting information can be downloaded at: https://www.mdpi.com/article/10.3390/ijerph19169920/s1.
