# Supplementary material for: Reduced Vastus Medialis/Lateralis EMG Ratio in Volleyballers with Chronic Knee Pain on Sports-Specific Surfaces: A Pilot Study
Source: Int J Environ Res Public Health. 2022 Aug 11;19(16):9920. doi: 10.3390/ijerph19169920 (PMC9408285; doi:10.3390/ijerph19169920)
Supplement: Supplementary file 1 [file ijerph-19-09920-s001.zip › ijerph-1803292-supplementary.pdf]

International Journal of
Environmental Research and Public Health

MDPI

Supplementary Materials

Appendix S1: Impulse of leading and providing leg in injured and healthy athletes

Raw values of the leading and providing leg on the three different surfaces in the horizontal landing phase of a spike jump are displayed in the following figure (Figure S1). Impulse was calculated from foot contact until takeoff.

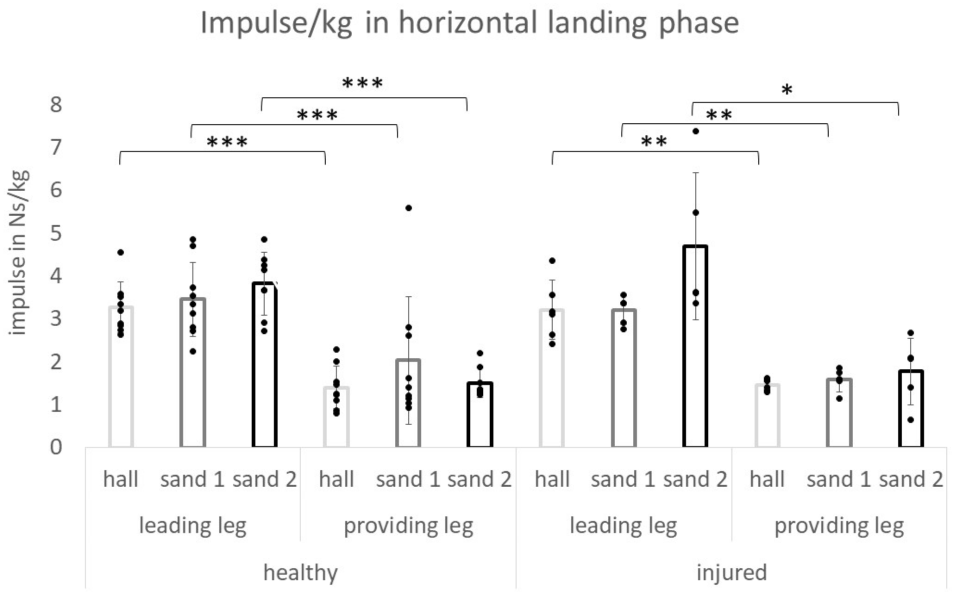

Figure S1. Impulse of leading and providing leg in the horizontal landing phase. Athletes were separated into injured and healthy groups. Impulse for leading leg was higher than providing leg, but there were no obvious group differences. * indicates $p < 0.1$; ** indicates $p < 0.05$; *** indicates $p < 0.001$.

In the next figure (Figure S2) impulse in landing is displayed. Impulse was calculated over a 300 ms window beginning from first foot contact after the jump.

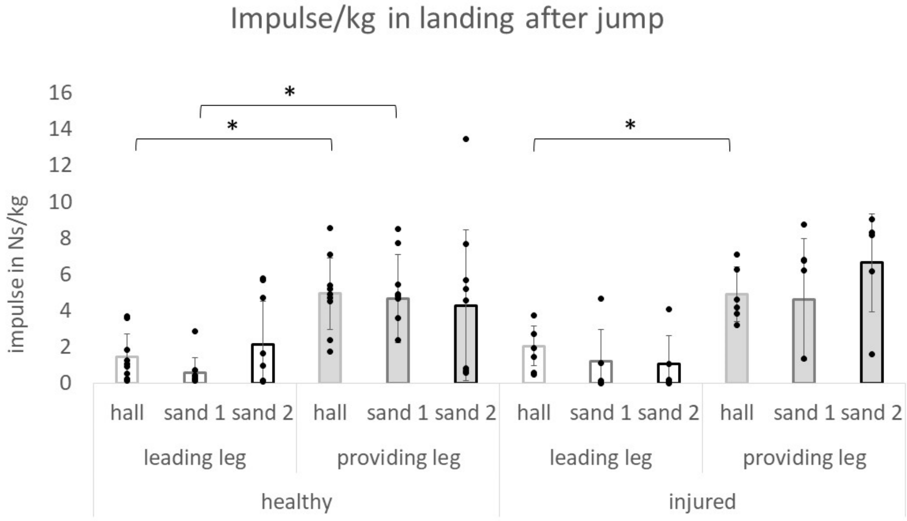

Figure S2. Impulse of leading and providing leg in the landing phase after the jump. Athletes were seperated into injured and healthy groups. Impulse for providing leg was higher than leading leg, but there were no obvious group differences. * indicates $p < 0.5$.

Appendix S2: Injured versus healthy athletes

Raw values of the VM/VL ratio of the CMJs and spike jumps of injured and healthy athletes are displayed in the following tables. Table S1 includes the VM/VL ratios of the unilaterally injured athletes, in whom the injury always occurred in the leading leg, and the two excluded bilaterally injured athletes (m/f).

Table S1. VM/VL ratios of injured and healthy players performing a CMJ on a hard surface, sand surface 1, and sand surface 2. The male injured athlete is indicated with [m].

	subject	injured			healthy		
		hard	sand 1	sand 2	hard	sand 1	sand 2
unilateral	1	0,82	0,91	1,01	1,67	1,47	1,46
	2	1,04	1,00	0,45	1,02	1,25	1,19
	3	0,69	0,50	0,57	0,69	0,98	3,19
	4	0,54	0,75	0,66	2,24	2,49	1,46
	5	0,65			0,61	1,16	0,49
	6	0,78			1,17	0,64	1,66
	7					1,61	1,73
		injured leading leg			injured providing leg		
bilateral	1	0,35	0,46	0,33	0,28	0,53	0,28
	2 [m]	0,81	0,89	1,03	0,78	0,61	0,66

Abbreviations: m=male

Table S2. VM/VL ratio of injured and healthy players performing a spike jump on a hard surface, sand surface 1, and sand surface 2. The male injured athlete is indicated with [m].

	subject	injured			healthy		
		hard	sand 1	sand 2	hard	sand 1	sand 2
unilateral	1	0,89	0,81	1,03	1,99	1,50	1,99
	2	1,02	1,02	0,94	1,09	0,97	1,45
	3	0,65	0,63	0,52	1,13	1,73	1,33
	4	0,59	0,55	0,63	0,76	0,93	2,06
	5	0,66	0,57	0,93	2,13	3,12	0,92
	6	0,97	1,12		0,86	1,30	1,64
	7				1,34	1,73	1,55
		injured leading leg			injured providing leg		
bilateral	1	0,43			0,22		
	2 [m]	0,70	0,74	0,73	0,88	0,73	0,94

Abbreviations: m=male

Appendix S3: Spearman's correlation for neuromuscular control

Jump tasks and surfaces

Raw values of the VM/VL ratios of CMJs and spike jumps on hard, sand 1, and sand 2 surfaces are displayed in Appendix 4. The correlations between CMJs and spikes are displayed in the left figure and the correlations between surfaces are in the right (Figure S3).

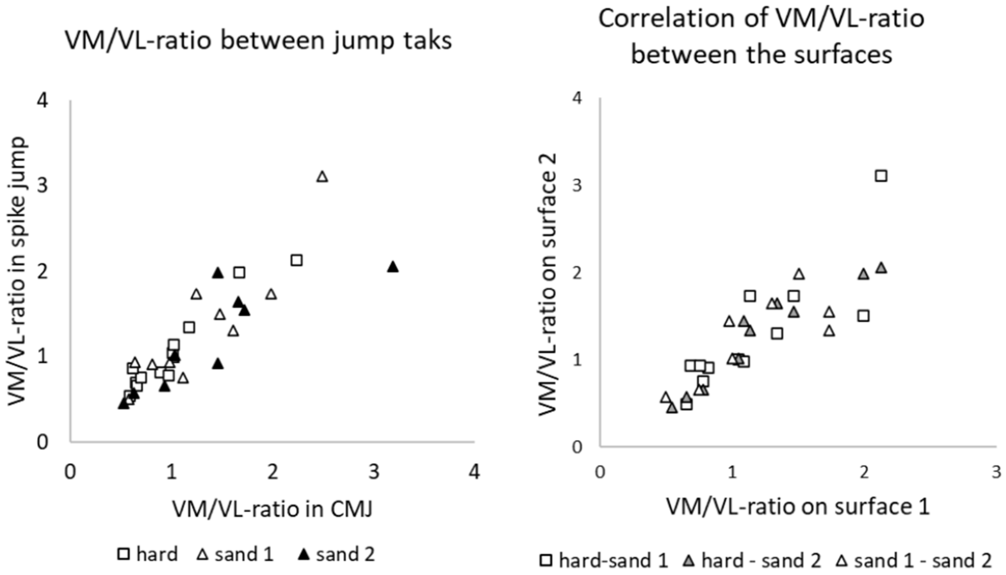

Figure S3. VM/VL ratios in CMJs and spike jumps on the three surfaces: hard surface, sand 1, and sand 2. On the left side, a comparison is made between the jump tasks and, on the right side, between surfaces.

Appendix S4: Bland–Altman analysis for intra-individual comparisons, jump tasks, and surfaces

In this appendix, the raw values and the Bland–Altman plots are presented for intra-individual comparisons, jump tasks, and surfaces.

Intra-individual differences

Intra-individual differences were tested with Bland-Altman analysis in CMJs and spike jumps. We compared the providing leg to the leading leg. Due to the low sample size, significant differences could not be calculated. Overall, 17 jumps were used for intra-individual comparisons for CMJs and 14 for spike jumps including all three surfaces. Raw values are displayed in Table S3.

Table S3. Raw values of VM/VL ratios in the injured athletes with the injured leading leg and uninjured healthy leg.

	jump	subject	hard		sand 1		sand 2	
			leading	providing	leading	providing	leading	providing
CMJ	unilateral	1	0,89	0,79	0,81	0,63	1,03	0,93
		2	1,02	0,70	1,02	0,78	0,94	0,61
		3	0,65	0,34	0,63	0,44	0,52	0,95
		4	0,59	0,82	0,55	0,98	0,63	1,67
		5	0,66	1,33	0,57	1,54	0,93	1,26
		6	0,97	1,37	1,12	1,22		
	bilateral	1	0,35	0,28	0,46	0,53	0,33	0,28
		2[m]	0,81	0,78	0,89	0,61	1,03	0,66
spike	unilateral	1	0,82	0,60	0,91	1,47	1,01	0,99
		2	1,04	0,66	0,99	1,03	0,45	0,88
		3	0,69	0,46	0,50	1,34	0,57	2,22
		4	0,54	0,81	0,75	1,65	0,66	1,08
		5	0,65	1,07				
		6	0,78	1,11				
	bilateral	1	0,43	0,22				
		2[m]	0,70	0,88	0,74	0,73	0,73	0,94

Abbreviations: m=male

Results of the Bland–Altman analysis are displayed in Figure S4. The gray crosses represent the CMJ and the black crosses, the spike jump. The VM/VL ratios of the injured leading and uninjured providing leg were almost equal on the hard surface for both jumps (difference in $mean_{CMJ} = 0.1$; difference in $mean_{spike} = 0.03$). Differences in the mean values of VM/VL ratios in the uninvolved compared with the injured limb in CMJs were higher on sand than in the hard surface (sand 1 = +0.15; sand 2= 0.25). The highest intra-individual difference in the VM/VL ratio was in the spike jump on both sand surfaces ($bias_{spike\_sand1} = 0.59$; $bias_{spike\_sand2} = 0.6$), where the VM/VL ratio in the uninjured providing leg was higher than in the injured leading leg. The standard deviation of the healthy limb was higher than that of the injured limb. In contrast, in the bilaterally injured athletes, the injured providing limb tended to have an even lower VM/VL ratio than the injured leading limb in CMJs and spike jumps represented by the triangles with negative values in the Bland–Altman plots.

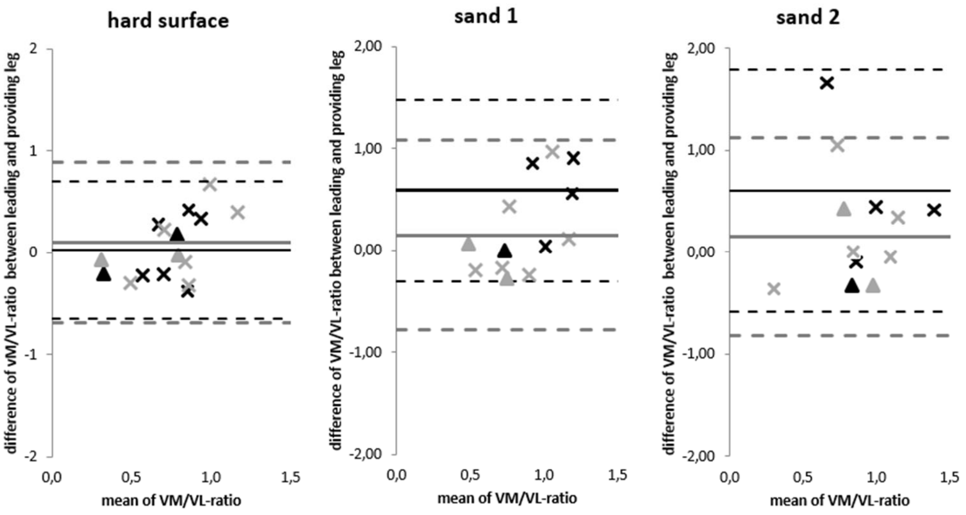

Figure S4. Bland–Altman plots of VM/VL ratios of leading and providing leg in the injured athletes. X indicates unilaterally injured athletes, Δ indicates bilaterally injured. Gray represents CMJ and black spike jump.

Jump tasks

The raw values and Bland–Altman analysis for the comparison between CMJs and spike jumps are presented in the following table and figure. The injured athletes are marked in gray.

Table S4. Raw values of VM/VL ratios in the injured (gray) and healthy (black) athletes for CMJs and spike jumps in hard, sand 1, and sand 2 surfaces. Table includes results of bilaterally injured athletes as well, even though they were not included in the analysis in the original paper.

subject	hard surface		sand surface 1		sand surface 2	
	CMJ	spike	CMJ	spike	CMJ	spike
unilateral 1	0,89	0,82	0,81	0,91	1,03	1,01
unilateral 2	1,02	1,04	1,02	1,00	0,52	0,45
unilateral 3	0,65	0,69	0,63	0,93	0,63	0,57
unilateral 4	0,59	0,54	0,57	0,50	0,93	0,66
unilateral 5	0,66	0,65	1,12	0,75		
unilateral 6	0,97	0,78				
bilateral 1[l]	0,35	0,43				
bilateral 1[p]	0,28	0,22				
bilateral 1[lm]	0,81	0,70	0,89	0,74	1,03	0,73
bilateral 1[pm]	0,78	0,88	0,61	0,73	0,88	0,94
healthy 1	1,67	1,99	1,25	1,73	1,46	0,92
healthy 2	1,02	1,13	0,98	0,93	1,66	1,64
healthy 3	0,69	0,76	2,49	3,12	1,73	1,55
healthy 4	2,24	2,13	1,61	1,30	1,46	1,99
healthy 5	0,61	0,86	1,98	1,73	3,19	2,06
healthy 6	1,17	1,34	1,47	1,50		

Abbreviations: l = leading leg
p= providing leg
m=male

Results of the Bland–Altman analysis are displayed in Figure S5. The gray crosses represent the injured athletes. The triangles are the bilaterally injured athletes. Each triangle represents the leading or providing leg. On all three surfaces, the standard deviations between the jumps of the injured athletes were lower in comparison to the healthy athletes. The ranges are marked in the figure with dotted lines. The exact values are displayed in the main text. The differences in the mean values and standard deviations for both limbs of the bilaterally injured athletes were low as well but were not included in the final analysis.

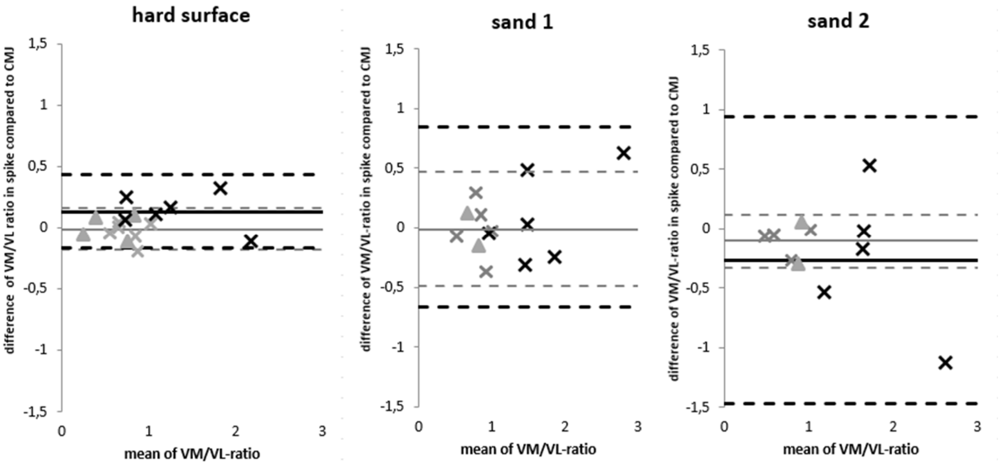

Figure S5. Bland–Altman plots of VM/VL ratios of leading leg of the healthy (black) and injured (gray) athletes between CMJs and spike jumps on the three different surfaces. X indicates leading leg of unilaterally injured or healthy athletes, and Δ indicates the leading and providing leg of the bilaterally injured athletes.

Surface

The raw values and Bland–Altman analysis for comparison of VM/VL ratios in spike jumps on the three surfaces are displayed in the following table and figure.

Table S5. Raw values of VM/VL ratio for comparing surfaces.

subject	hard surface	sand 1	hard surface	sand 2	sand 1	sand 2
unilateral 1	0,82	0,91	1,04	1,01	1,00	1,01
unilateral 2	1,04	1,00	0,54	0,45	0,50	0,57
unilateral 3	0,69	0,93	0,65	0,57	0,75	0,66
unilateral 4	0,65	0,50	0,78	0,66	1,50	1,99
unilateral 5	0,78	0,75	1,99	1,99	0,97	1,45
bilateral 1[lm]	0,70	0,74	0,70	0,73	0,74	0,73
bilateral 1[pm]	0,88	0,73	0,88	0,94	0,73	0,94
healthy 1	1,99	1,50	1,09	1,45	1,73	1,33
healthy 2	1,09	0,97	1,13	1,33	1,30	1,64
healthy 3	1,13	1,73	2,13	2,06	1,73	1,55
healthy 4	0,76	0,93	1,34	1,64		
healthy 5	2,13	3,12	1,47	1,55		
healthy 6	1,34	1,30				
healthy 7	1,47	1,73				

Abbreviations: l = leading leg
p= providing leg
m=male

Results of the Bland–Altman analysis are displayed in Figure S6. Gray crosses represent injured athletes, and black crosses indicate healthy athletes. Injured athletes had lower differences in mean values between surfaces than healthy athletes. The differences in standard deviations were much lower in injured athletes than in healthy controls. The exact values are provided in the main text. Differences in standard deviations were also low on both limbs for the bilaterally injured athletes.

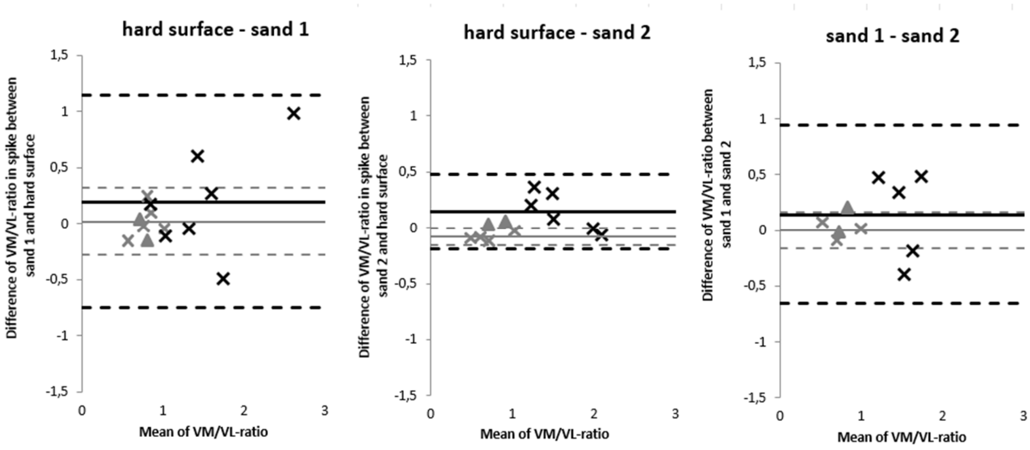

Figure S6. Bland–Altman plots of VM/VL ratio of leading leg of the healthy (black) and injured (gray) athletes on the three different surfaces. X indicates leading leg of unilaterally injured or healthy athletes, and Δ indicates the leading and providing leg of the bilaterally injured athletes.
